# Personalized medicine: a patient - centered paradigm

**DOI:** 10.1186/1479-5876-9-206

**Published:** 2011-12-01

**Authors:** Lotfi Chouchane, Ravinder Mamtani, Ashraf Dallol, Javaid I Sheikh

**Affiliations:** 1 Genetic Medicine Department, Weill Cornell Medical College in Qatar, Qatar Foundation, Education City, P.O. Box 24144, Doha, Qatar; 2 Public and Global Health Department of Weill Cornell Medical College in Qatar, Qatar Foundation, Education City, P.O. Box 24144, Doha, Qatar; 3 Center of Excellence in Genomic Medicine Research (CEGMR), King Fahad Medical Research Center, King Abdulaziz University, P.O. Box 80216, Jeddah 21589, Saudi Arabia; 4 Office of the Dean, Weill Cornell Medical College in Qatar, Qatar Foundation, Education City, P.O. Box 24144, Doha, Qatar

## 

An editorial about personalized medicine should perhaps start with a definition. Although several versions of such definition exist, we pay homage here to the oldest definition reported in modern medical literature. Sir William Osler (1849-1919) recognized that "variability is the law of life, and as no two faces are the same, so no two bodies are alike, and no two individuals react alike and behave alike under the abnormal conditions we know as disease". Modern day medicine recognized this fact and implemented its ethos since inception of its practice separating it from a general "one-size-fits-all" approach. A medical doctor would ask the patient about his/her suffering and prescribe a treatment suited to the patient's condition. Individualized evaluation and treatments, which include history taking, focused examination and specific laboratory and medical tests have now become routine in day-to-day medical practice. Personalized medicine, takes into account the needs of individual patients, and provides custom-tailored therapeutic approaches.

More recently, modifying life style approaches as part of a broad preventive medicine orientation, are gaining popularity and yielding positive results. Weight management, smoking cessation and healthy diet are well-established preventive strategies that have a contributed a great deal in reducing mortality associated with chronic diseases. But there are challenges. For example, losing weight is easy. But, maintaining it at an optimum level is a challenge!

Similarly, limits of existing diagnostic and therapeutic strategies are becoming well-known. Despite all the impressive advances in imaging technology, advent of new medical diagnostics, and burgeoning of therapeutic interventions, the widespread prevalence of disability and premature mortality associated with chronic conditions such as diabetes, cancer and heart disease continues to frustrate scientists and clinicians alike. There are also many unanswered questions. Here is one such question. Why two patients with exactly the same diagnosis and identical test results respond differently to the same treatment.

Have we reached a glass ceiling? Are we limited in our scientific understanding of disease and health? Rapid advances in genotyping and genomics might shed some light. Let us look at example of the oral anticoagulant drug Warfarin that is used for the long-term management of thromboembolic events. Studies have shown that of over 21 million patients, who are on Warfarin in the USA, some suffer from its adverse effects [1] and others don't. Why? Research has shown that there is a variant nucleotide in the Cytochrome P450 CYP2C9, which is responsible for this variation observed in the drug response [2]. Other genetic variations that alter the personal response to Warfarin also exist in the Vitamin K epoxide reductase complex protein 1 (VKORC1) [3]. The U.S. Food and Drug Administration (FDA) has recognized and acknowledged the importance of genotyping CYP2C9 and VKORC1 during Warfarin treatment [4,5]. In doing so, it has given the field of genomics a tremendous boost.

Let us take a look at another example. Trastuzumab is a very effective drug for breast cancer treatment. However, only 10-20% of the breast cancer patients can benefit from it. This is due to the fact that Trastuzumab is based on monoclonal antibodies targeting the Epidermal Growth Factor Receptor (HER2/neu/EGFR) [6]. Therefore only patients with amplification (multiple copies) of HER2/neu/EGFR will respond to this treatment. The availability of Trastuzumab has created a research drive at a frantic pace, to standardize the detection of HER2/neu/EGFR amplification, for which several methods are now available.

The utility of genomics in personalized medicine is gaining popularity. Its potential for predicting disease occurrence is receiving worldwide attention and illustrated by examining the relationship between certain allele's and cancer risk. For example, the presence of mutant BRCA1 or BRCA2 allele substantially increases the risk of breast and/or ovarian cancer. Similarly, the presence of certain variant single nucleotide polymorphisms (SNPs, e.g. FGFR2) also significantly escalates the probability of developing breast cancer. Specific SNPs have also been identified which are associated with increased risk of diabetes, rheumatoid arthritis or chronic heart disease as well as other multi-factorial diseases. This list is continuously being updated as additional SNPs are being identified from a wide range of promising genome-wide association studies, which are underway throughout the world. Technology aimed at predicting disease and health outcomes is gaining momentum.

The promise of genomic evaluation as an integral component of personalized medicine is fast becoming a reality in many nations around the world. The recent announcement of the formation of the Genomic Cancer Care Alliance between one of the biggest providers of next-generation sequencing solutions, Life Technologies, and leading research centers such as Fox Chase Cancer Center, Scripps Genomic Medicine, and the Translational Genomics Research Institute (TGen) is an illustration of that reality. The goal of this alliance is to launch a pilot study aimed at determining whether whole-genome sequencing can positively affect the treatment decisions across a number of cancers with limited treatment options. Research laboratories with access to the high-throughput sequencing technology are already implementing Whole-Exome Seq in the identification of genetic causes of congenital abnormalities, such as congenital hearing loss.

Current concept of 'Personalized medicine' approach thus incorporates the traditional assessment methods, genotyping, and genomic evaluation in predicting disease risk and treatment outcomes. Additionally, it encourages patients to participate in their own care: participative component of personalized medicine. Research has shown that patients who participate in their own care have better outcomes than patients who don't [7].

Most health care 'gurus' agree that health care will become more person-specific in its approach, and will be driven by the patients' felt needs, their perceptions about health and disease and their behavior. Taking into account the patient's behavior and other factors surrounding the doctor-patient relationship in managing disease and illness is vital, and will become more important in years ahead.

It is well known that human factors, which significantly improve disease outcomes, are many [8]. Healing words, pleasant environment, and family and social support, to name a few, are well known examples of such factors. Patient feelings and attitudes also matter. Positive attitude and feelings result in better outcomes. Negative feelings and hopelessness, on the other hand, can have detrimental effects. There is now evidence, for example, that hopelessness accelerates carotid atherosclerosis [9].

Interaction between the doctor and the patient is vital in the overall healing response. Empathy, caring and helping patients cope with their suffering have a real impact on patient outcomes.

There are many scientists who concur that many human factors, placebo or context factors as some may call them [8], described above may be operating via psychoneuroimmunology paradigm [10], which is "the complex interrelationship between the mind or psychology, the brain, the immune system and general health". A recent study showed an association between genetically controlled amygdala activity and placebo-induced relief from anxiety. This is a striking observation and will no doubt lead to additional research initiatives on this subject [11] Soon, we will see scientists beginning to identify allele's and SNP which guide human behavior and factors (placebo and context effects). That information could be immensely helpful in optimizing healing responses in certain individuals.

The genomics technology is advancing at a rapid pace. The cost of sequencing whole human genomes is now within reach of most research laboratories. As the technology continues to grow and advance we need to be mindful of the challenges and questions that the upcoming discipline of 'personalized medicine' is likely to present in times ahead. The key question for a health care provider is who will pay the high for the use of personalized genomics? In the U.S.A, medical insurance companies are so far resisting re-imbursements for routine genetic testing delaying the implementation of personalized medicine. It remains to be seen how such technology will be paid for in other countries like the United Kingdom where the health service is largely funded by the public sector (National Health Service, NHS).

Here is another challenging question. How likely is it that individual genome information may be used to discriminate against people with negative health and personal traits? This could be a serious ethical issue that the law and policy makers may have to grapple with in times ahead. Protecting the confidentiality of the genomic information will also be of concern.

Despite some of the concerns noted above, personalized medicine is the way forward. It is a melding of traditional (e.g., personalized history, examination, and laboratory tests) and novel approaches, e.g., genotyping, genomic evaluations). It uses the science of prediction, principles of modern therapeutics and prevention, and optimizes active participation of patients in their own care. Treating the patient as a person (with his/her human attributes) and not just their illness is also an essential element of this approach. This wholesome and person-centered approach to health care should improve outcomes, reduce morbidity and mortality, and at the same time alleviate pain and human suffering commonly associated with chronic illnesses such as cancer and heart disease.
